# 

**Published:** 1904-01-23

**Authors:** 


CONTENTS.

**The Health of London** - 285

**The Freedom of the Profession** - 286

**Annotations** - 287

**Notes and News** - 288

**Hospital Clinics and Medical Progress—**

What We Owe to Experiments on Animals - 289

The Early Diagnosis of Perforation in Typhoid Fever - 290

The Temperature in Shock - 291

The Mortality of Appendicitis - 291

PROGRESS IN SURGERY OF THE INTESTINES - 292

PROGRESS OF SURGERY - 293

PROGRESS IN FEVERS - 294

PROGRESS IN SURGERY OF THE STOMACH - 295

**New Appliances and Things Medical** - 295

**The Book World of Medicine and Science** - 296

**Modern Sociology** - 297

**Hospital Administration—**

St. Bartholomew's Hospital - 299

The Royal Waterloo Hospital for Children and Women - 304

The Story of the Insane from Year to Year - 304

**Editor's Letter-Box** - 305

**The Student of Medicine** - 306

*NURSING SECTION.*

**Notes on News from the Nursing World—** The Matron of Osborne House—Army Nurses asked to Volunteer for Service—Queen Alexandra's Imperial Military Nursing Service—The Colonial Nursing Association—The Matron of Johannesburg Hospital and her Nurses—Royal British Nurses' Association—Registered Nurses in America—The Vacancy at Norfolk and Norwich Hospital—Meals in a Bedroom at a Workhouse Infirmary—Belfast Nurses' Home—The Ideal Asylum Nurse—Dereliction of Duty at a Brighton Hospital—Friction at West Ham Workhouse—An Age Question at Lewes—Progress at Newport Union Infirmary—The New Institute at Welshpool—The Irish Nurses' Association—The Nurses of Taunton Hospital—Short Items - 225-228
**The Nursing Outlook** - 229
**Sir William Bennett on Modern Nursing in Private Practice** - 230
**Queen Alexandra's Imperial Military Nursing Service** - 232
**The Application of Hot Irrigation to a Leg with a Compound Fracture** - 232
**A Function at the North-Eastern Hospital for Children** - 233
**St. Bartholomew's Hospital, Rochester** - 233
**Everybody's Opinion** - 234
**Appointments** - 235
**The Nurses' Bookshelf** - 235
**Presentations** - 235
**Echoes from the Outside World** - 236
**A Book and its Story** - 237
**Notes and Queries** - 238
**For Reading to the Sick** - 238

